# The importance of integrating flexible learning methods (audio‐visual animation vs. visual pamphlet) to enhance awareness, perspectives, and practices in preventing lower back pain in nurses. A quasi‐experimental study

**DOI:** 10.1002/hsr2.70127

**Published:** 2024-10-15

**Authors:** Nahid Zarifsanaiey, Zahra Yazdani, Zahra Karimian, Hadi Raeisi Shahraki

**Affiliations:** ^1^ Department of E‐Learning, Virtual School and Center of Excellence for E‐Learning in Medical Sciences Shiraz University of Medical Sciences Shiraz Iran; ^2^ Department of Epidemiology and Biostatistics, Faculty of Health Shahrekord University of Medical Sciences Shahrekord Iran

**Keywords:** audio‐visual, awareness, flexible learning, low back pain, practice, visual pamphlet

## Abstract

**Background and Aims:**

Nurses are highly susceptible to developing low back pain (LBP), which is considered a common occupational hazard. The present study investigated the efficacy of flexible learning methods on the nurses' awareness, perspectives, and practice regarding the prevention of LBP.

**Methods:**

In pre‐test posttest quasi‐experimental study conducted from June to December 2019, 153 eligible nurses working in three hospitals were participated. Researchers divided the hospitals into three groups with similar numbers of participants (around 55 each) using a random process. One group received an educational intervention using an audio‐visual animation, another group got a visual pamphlet, and the last group served as a control with no intervention. All nurses completed a validated questionnaire designed by the researchers to assess their awareness, perspectives, and practices related to preventing lower back pain. The questionnaire was given three times: before the intervention, 1 week after, and 4 weeks after.

**Results:**

The study found that participants in the audio‐visual and visual pamphlet groups scored significantly higher on awareness, perspective, and practice measures compared to the control group. Interestingly, the visual pamphlet group showed even greater awareness and practice scores compared to the audio‐visual group (*p* < 0.001). However, the audio‐visual group achieved a higher perspective score compared to the visual pamphlet group (*p* < 0.001).

**Conclusion:**

Nurses' perspective, awareness, and practice towards LBP prevention can be improved by blending the visual pamphlet and audio‐visual animation.

## 1 BACKGROUND

Nurses are highly susceptible to low back pain (LBP), a prevalent issue affecting their well‐being and work performance.^1^ Several studies have identified various risk factors for LBP among healthcare workers, including long work hours.^2^ These long hours, combined with physically demanding tasks like lifting patients and prolonged standing^3^, ^4^ contribute significantly to LBP in nurses.

This pain can have a cascading effect on healthcare delivery. Nurses experiencing LBP may be more likely to miss work due to absenteeism, make medication errors, or even consider early retirement or career changes.^3^

Implementing targeted training programs specifically for nurses can be a powerful tool. These programs can equip nurses with the knowledge and skills needed to prevent LBP in the first place. A healthier workforce translates to a more efficient healthcare system, ultimately leading to improved patient care. Therefore, selecting the most effective educational method is crucial for maximizing learning outcomes and fostering long‐term behavior changes that promote LBP prevention.^4^

Traditional education often takes a one‐size‐fits‐all approach. However, the rise of flexible learning offers a more adaptable experience for busy professionals like nurses, catering to individual learning styles and preferences.^5^

Social cognitive theory (SCT) emphasizes the interaction between personal factors, environmental influences, and behavior. It posits that individuals learn through observation, modeling, and reinforcement. SCT can be applied to flexible learning programs by incorporating elements that promote self‐paced learning, empowering nurses to regulate their learning process.^6^ Modern nursing education embraces digital technologies, offering flexible, self‐paced learning experiences. Prerecorded video lectures with clear explanations, animations, and demonstrations cater to auditory and visual learners, while animations can benefit kinesthetic learners.^7^

On the other hand, some nurses prefer a more tangible approach. Visual pamphlets offer a quick reference guide or a supplementary learning tool. Images, infographics, and concise text visually represent key LBP prevention strategies. The portability of digital pamphlets allows nurses to download them for access during downtime or patient interactions. Printed pamphlets offer the same convenience without relying on internet connectivity.^8^

This diverse approach caters to various learning styles. By combining audio‐visuals and visual pamphlets, flexible learning creates a well‐rounded educational experience for busy nurses with individual learning styles and specific needs for LBP prevention.

Several studies in healthcare settings have investigated the effectiveness of audio‐visual and pamphlet methods in knowledge retention and skill development. The majority of the research suggests that audio‐visual methods have a more significant impact on knowledge and skills in healthcare settings (e.g.,^9^, ^10^). However, the findings are not always unanimous. A 2019 systematic review by Zarifsanaiey et al.^10^ analyzed the effectiveness of various educational methods for patients, highlighting the importance of tailoring the approach to specific needs. Multiple studies have shown the effectiveness of educational interventions in improving knowledge. For instance, research by Handayani & Yulaikah^11^ demonstrated that both educational software and booklets can be successful in enhancing pregnant women's understanding of labor pain management.

Yohana et al.^12^ reported on a small number of studies where no significant difference was found between audio‐visual and pamphlet methods of high school teachers about prevention of health‐risk behaviors. However, the influence of these methods on nurses' LBP prevention awareness, perspectives, and practices remains under‐investigated.

Nurses are particularly susceptible to LBP, highlighting the need for effective training programs. Current LBP prevention education might lack engaging formats or accessibility, potentially hindering knowledge retention and application.^13^ Notably, a similar study has not been conducted at Shiraz University of Medical Sciences (SUMS), creating a significant knowledge gap.

Therefore, this study aims to evaluate the efficacy of a flexible learning program using audio‐visual animation and visual pamphlets to enhance nurses' awareness, perspectives, and practices regarding LBP prevention.

## 2 MATERIAL AND METHODS

### 2.1 Design

In this pre‐test posttest quasi‐experimental study, 180 nurses were enrolled from three hospitals associated with SUMS in Shiraz, Iran from June to December 2019.

### 2.2 Inclusion/Exclusion criteria

**Figure 1:**
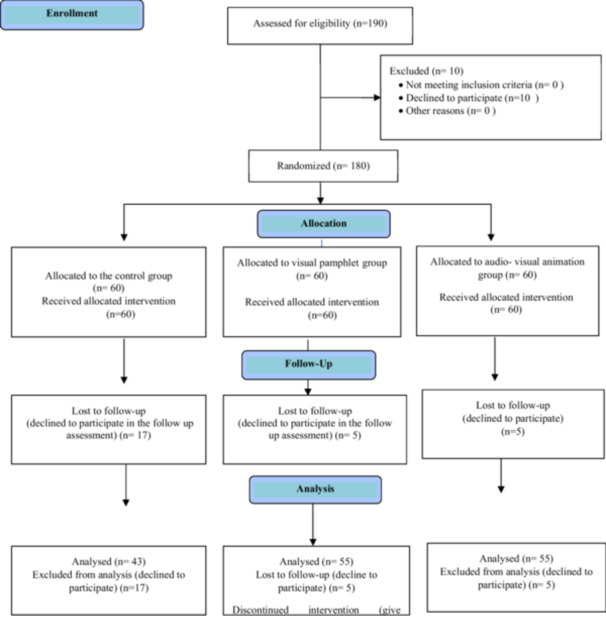
The participants' recruitment flow diagram.

The inclusion criteria encompassed nurses employed in those hospitals, possessing at least a bachelor's degree in nursing, expressing a willingness to participate, providing informed permission, and demonstrating a basic proficiency in computer skills. The study included participants who could actively participate, provided informed consent, and possessed basic computer skills. However, to ensure the validity of the results, certain individuals were excluded: those with congenital abnormalities, a history of back surgery or trauma, pregnancy, or severe osteoporosis. Additionally, participants who withdrew their cooperation or took an extended leave of absence during the study were omitted from the final analysis (Figure 1).

### 2.3 Educational interventions

After obtaining the required approvals from the university research committee and the directors of public hospitals in Shiraz, the investigators commenced the study by presenting the participants with a brief overview of the initiative and securing their written consent. Eligible nurses who agreed to participate signed the informed consent form and completed a pre‐test questionnaire evaluating their awareness, perspectives, and practices.

A. ***Interventional groups:*** In this intervention study, educational content was developed and two intervention and one‐control groups were implemented. The intervention groups received LBP educational content through audio‐visual animation (Group 1) and visual pamphlet (Group 2) methods, while no intervention was given to the Wait‐list control group. The aim of this study was to enhance nurses' awareness, perspectives, and practice towards prevention of LBP.
  A professor of rehabilitation and a nurse specialist in e‐learning were responsible for developing educational content. Researchers meticulously reviewed books and credible published articles to gather evidence‐based LBP prevention methods. These methods included proper posture for sitting, sleeping, walking, using a computer, lifting equipment, moving patients, and exercising.^13^
  The educational materials themselves were designed based on self‐directed and multimedia learning principles. These theories posit that learners retain information more effectively when they can engage with the content at their own pace and convenience.^9^ The educational intervention for this group consisted of a 30‐min animated video. This animation incorporated text, images, and narration to deliver information about LBP prevention in a clear and engaging way.
  The visual pamphlet, which included written and visual content, was converted into a colorful PDF to produce an appealing pamphlet with illustrations across 15 A5‐size pages. The topics remained consistent in both the audio‐visual and visual pamphlets. Both guides underwent technical and content evaluation by two experts, and the final versions were accessible to participants through WhatsApp, providing flexible and self‐directed learning options. Following the pre‐test, participants were randomized into two intervention groups: Group 1 received an audio‐visual animation, and Group 2 received a visual pamphlet. Each group was assigned to a separate, closed social media platform group (e.g., WhatsApp).
  Within their assigned groups, participants received access to the educational content relevant to their intervention (audio‐visual animation or visual pamphlet). They were encouraged to actively engage with the content and with each other by sharing their experiences, participating in discussions about LBP prevention strategies, and posing questions. Researchers monitored the group discussions to ensure a safe and respectful environment. They addressed participant inquiries, provided summaries of key topics to facilitate knowledge retention, and may have offered additional prompts to stimulate discussion. Over a 1‐week period, participants had the flexibility to review the content at their own pace, ask questions, and interact with their peers within the secure social media platform. The three groups completed a questionnaire 1 week and 4 weeks after the end of the training to assess the impact of the intervention on their awareness, perspectives, and practice towards the prevention of LBP.
B. ***The control group:*** It is important to note that the control group was not given the instructional materials until the end of the investigation, and thus did not have any interventions.

### 2.4 Instruments

The nurses' awareness, Perspectives, and Practices to prevent LBP were evaluated at baseline, one, and 4 weeks after interventions. Initially, qualified nurses received the link to online questionnaires via WhatsApp 24 h after agreeing to participate. Those who did not respond to the questionnaires were contacted and encouraged to complete them. It is crucial to highlight that only one follow‐up attempt per participant was conducted. The data collection tool was a researcher‐made questionnaire included:

- ***Demographic information & employment background.***
- ***Awareness*** This aspect examined the awareness of nurses regarding preventive measures related LBP, which consisted of ten multiple‐choice questions. Each correct answer was awarded one point, whereas an incorrect answer received a score of zero, with a total score ranging from zero to 10 points.
- ***Perspectives***: This aspect assessed nurses' viewpoints on preventive measures for LBP using an 18‐question Likert scale ranging from “strongly agree” (6 points) to “strongly disagree” (1 point). A higher score on this scale indicates a more positive and supportive attitude towards LBP prevention strategies.
- ***Practices***: This dimension evaluated nurses' actual implementation of LBP prevention practices through a 19‐question Likert scale, ranging from “always” (6 points) to “never” (1 point). In this section, a higher score reflects a greater frequency of adherence to recommended LBP prevention practices in the workplace.

To ensure the questionnaire accurately measured nurses' awareness, perspectives, and practices regarding LBP prevention, the research team employed several validation techniques.

#### 2.4.1 Content validity

Twenty experts from nursing and rehabilitation fields reviewed the questionnaire for clarity, relevance, and appropriate language.

Construct Validity: Confirmatory factor analysis confirmed that the questionnaire effectively captured the intended constructs.

#### 2.4.2 Reliability

Test‐retest reliability was assessed among 60 nurses over a 4‐week period, demonstrating consistent results (r = 0.54, *p* < 0.001). Additionally, Cronbach's alpha and Composite Reliability (CR) values indicated strong internal consistency for both attitude (0.92 & 0.925) and practice (0.94 & 0.95) sections.

#### 2.4.3 Convergent validity

Average Variance Extracted (AVE) scores of 0.51 for perspective and 0.50 for practice confirmed that the questionnaire measured these concepts well.

#### 2.4.4 Divergent validity

The Fornell‐Larcker test further supported the distinctiveness of the measured constructs.

The questionnaire completion time was approximately 15 min. Participants received unique IDs for tracking purposes.

### 2.5 Sample size

To determine an appropriate sample size for this study, we employed a power analysis considering the following factors:

Preliminary data: Based on our pilot sample of 60 nurses, we observed a potential difference in the awareness scores between the audio‐visual animation and visual pamphlet groups (mean difference = 1.5, standard deviation = 0.1).

Statistical power: We aimed for a power of 0.80 to detect this effect size with a significance level of alpha = 0.05 (type I error) and a beta level of 0.10 (type II error).

Potential withdrawal: We anticipated a possible withdrawal rate and adjusted the initial sample size accordingly. Following this power analysis, the calculated sample size was determined to be 126 nurses. To account for potential participant withdrawal, we inflated the sample size to 190 participants.

To achieve the desired sample size, 130 nurses were recruited following a pilot study that included 60 participants (20 per group).

$$N=\frac{(s_{1}^{2}+s_{2}^{2}){{(Z}_{1-\frac{\alpha }{2}}+Z_{1-\beta })}^{2}}{{\left(\mu_{1}-\mu_{2}\right)}^{2}}$$

### 2.6 Randomization

Hospitals, rather than individual nurses, were chosen as the unit of randomization to prevent contamination between intervention and control groups.

A three‐stage process ensured a balanced and representative sample. First, three hospitals with similar characteristics (specialty, workload, nurse demographics) were selected using a convenience sampling method. These hospitals were then randomly assigned to either the audio‐visual animation, visual pamphlet, or control group using a random number table. Finally, within each hospital, three wards with comparable nurse workloads (surgery, orthopedics, emergency) were purposefully chosen. Nurses were then recruited until a target of 60 participants per hospital was reached (June–December 2019). An independent research assistant, not involved in the study methodology, conducted the randomization process.

### 2.7 Statistical methods

SPSS‐21 software was used to analyze the collected data following established SAMPL guidelines. Descriptive and inferential statistics were employed to understand the data set. Repeated‐measures analysis compared pre‐test and posttest results, ANOVA and ANCOVA with LSD post‐hoc tests assessed group differences, and the Chi‐square test evaluated relationships between categorical variable. In cases where a significant interaction between time and group was observed, separate repeated‐measures analyses were conducted to examine the effect of time within each group. This approach allowed for a more nuanced understanding of the data and the impact of the variables under investigation. The significance level was set at *p*‐value < 0.05, indicating that any results with a *p*‐value lower than this threshold were considered statistically significant. This threshold is commonly used in statistical analysis to determine whether the observed differences between groups or variables are likely due to chance or to a real effect.

### 2.8 Ethics

The study was carried out after receiving ethical approval from the local ethics committee of SUMS (1398.040) and coordinating with the teaching hospitals. The research team thoroughly explained the study's objectives to participants and obtained their written informed consent to participate. To safeguard anonymity, all questionnaires were left blank for participant names. A research assistant then coded the completed questionnaires to minimize the risk of errors during data entry. The participants were assured of the confidentiality of their data and that only aggregated statistics would be presented. The participants were also informed of their right to withdraw from the study at any time. In addition, the researchers confirm that all methods were carried out in accordance with relevant guidelines and regulations.

## 3 RESULTS

Out of 180 eligible nurses, 153 (85%) completed the study (43 in control, 55 in visual pamphlet, and 55 in audio‐visual animation groups). The remaining 27 participants (17, 5, and 5 from each group respectively) declined the follow‐up assessment and were excluded from the final analysis.

In this study, the demographic data of participants in both the intervention and control groups were found to be consistent, as shown in Table 1.

**Table 1:** Demographic Data of Nurses in the Intervention and Control Groups.

Variable	Subgroup	Control (*n* = 43)	Visual pamphlet (*n* = 55)	Audio‐visual animation (n = 55)	*p*‐value
Gender	Female	16 (37.2)	13 (23.6)	17 (30.9)	0.34
	Male	27 (62.8)	42 (76.4)	38 (69.1)	
Marital status^a^	Single	20 (46.5)	32 (58.2)	31 (56.4)	0.48
	Married	23 (53.5)	23 (41.8)	24 (43.6)	
Employment status	Contractual	7 (16.3)	12 (21.8)	15 (27.3)	0.36
	Corporate recruitment	4 (9.3)	4 (7.3)	8 (14.5)	
	Temporary	4 (9.3)	4 (7.3)	5 (9.1)	
	Government employment	19 (44.2)	15 (27.3)	13 (23.6)	
	Recruitment plan	9 (20.9)	20 (36.4)	14 (25.5)	
Work experience	<10 years	23 (53.5)	36 (65.4)	38 (69.1)	0.17
	10‐20 years	18 (41.9)	15 (27.3)	17 (30.9)	
	>20 years	2 (4.7)	4 (7.3)	0 (0.0)	
Weight		65.0 ± 9.4	65.4 ± 10.8	68.3 ± 13.9	0.28
BMI		166.4 ± 7.8	165.6 ± 7.1	166.6 ± 9.0	0.24

^a^ None of the research samples were in a state of divorce, separation, or widowhood.

### 3.1 Awareness score

**Figure 2:**
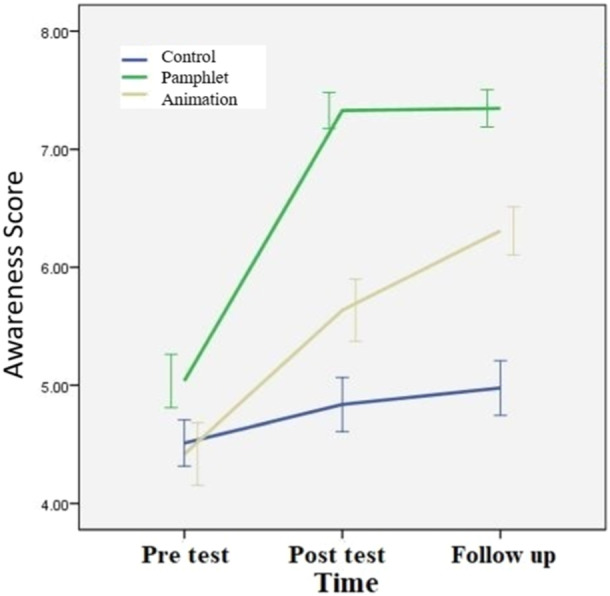
The trend of awareness score in different groups.

There were no significant differences in baseline LBP prevention awareness scores among the groups (*p* = 0.13). However, the visual pamphlet group demonstrated significantly greater awareness compared to both the control and animation groups at the 1‐week (*p* < 0.001) and 4‐week (*p* < 001) follow‐up assessments (Figure 2).

In addition, the results presented in Table 2 shows that although both educational methods could affect the awareness of the nurses, the visual pamphlet had a greater effect (*p* < 0.001) (Table 2).

**Table 2:** Comparative analysis of audio‐visual animation, visual pamphlet, and control groups in terms of awareness perspective, and practice, both between and within groups.

Variable	Group	Pretest	Posttest	Follow up	Time effect	Group effect	Time^a^ group effect
					*p*‐value^a^	*p*‐value^a^	*p*‐value^a^
Awareness	Control	4.5 ± 1.3	4.8 ± 1.5	5.0 ± 1.5	0.06	<0.001	<0.001
	Visual pamphlet	5.0 ± 1.7	7.3 ± 1.1	7.4 ± 1.2	<0.001		
	Audio‐visual animation	4.4 ± 2.0	5.6 ± 2.0	6.3 ± 1.5	<0.001		
Between group comparison *p* value		0.13^b^	<0.001	<0.001			
Perspective	Control	99.0 ± 14.5	96.2 ± 10.7	96.0 ± 10.2	0.03	0.11	<0.001
	Visual pamphlet	100.4 ± 16.4	94.2 ± 8.2	91.7 ± 7.4	<0.001		
	Audio‐visual animation	89.0 ± 15.5	93.0 ± 14.9	94.1 ± 12.8	0.002		
Between group comparison *p* value		0.001^b^	0.06^c^	<0.001^c^			
Practice	Control	70.1 ± 16.1	69.6 ± 14.3	69.4 ± 14.3	0.41	<0.001	<0.001
	Visual pamphlet	69.7 ± 19.7	80.1 ± 12.5	84.4 ± 14.4	<0.001		
	Audio‐visual animation	61.7 ± 18.1	66.9 ± 18.1	69.1 ± 15.8	<0.001		
Between group comparison *p* value		0.03^b^	<0.001^c^	<0.001^c^			

^a^ Repeated measurement analysis.

^b^ Independent *t*‐test.

^c^ Analysis of covariance (considering pre‐test as covariate).

### 3.2 Perspective score

Analysis of covariance (ANCOVA) was used to examine differences in LBP prevention perspectives between the groups at one and 4 weeks after the intervention. This analysis considered pre‐test scores to adjust for any potential baseline differences between the groups.

**Figure 3:**
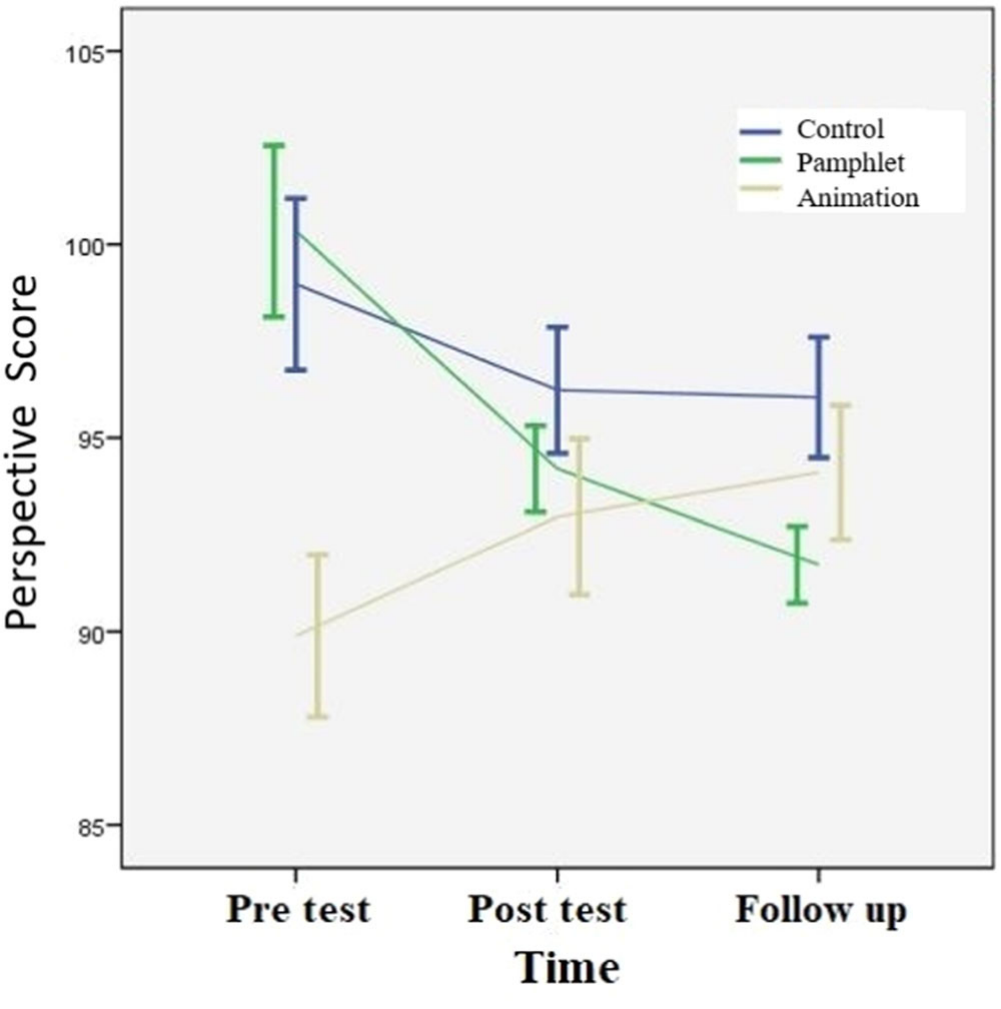
The trend of Perspective score in different groups.

One week after the intervention, there was no significant difference between the perspective scores for the various groups (*p* = 0.06). However, 4 weeks after the intervention, the visual pamphlet group's perspective score was substantially less than the control group's (*p* = 0.04), as shown in Figure 3.

Table 2 demonstrates a change in perspective scores over time within the intervention groups. While scores in the visual pamphlet group decreased, scores in the audio‐visual animation group conversely increased. Notably, the increase observed in the audio‐visual animation group was significantly greater than that in the visual pamphlet group (*p* < 0.001).

### 3.3 Practice score

**Figure 4:**
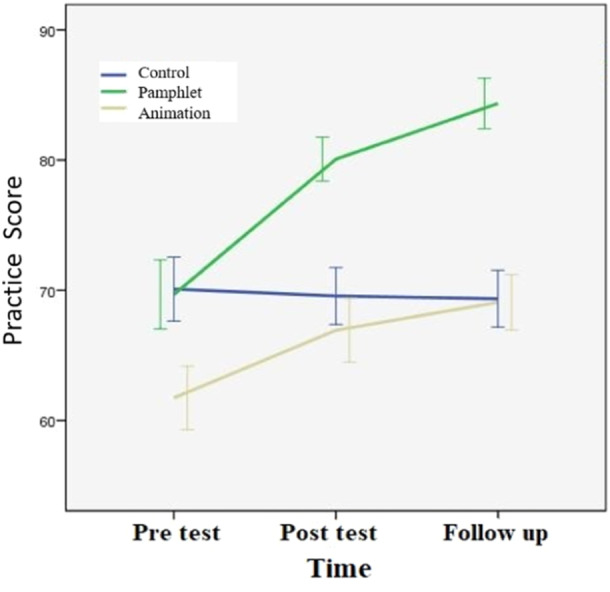
The trend of practice score in different groups.

Table 2 shows that both interventions led to significant improvements in LBP prevention practices. Nurses in both the visual pamphlet (*p* < 0.001) and audio‐visual animation groups (*p* < 0.001) demonstrated a notable increase in their average practice scores. It is worth noting that, following the intervention, both the visual pamphlet and audio‐visual animation groups experienced significant improvements in their perspectives, as shown in Table 2. However, the improvement observed in the Audio‐visual animation group was considerably higher than that observed in the visual pamphlet group (*p* < 0.001). Furthermore, this superiority was maintained even after controlling for the pre‐test score, indicating that the impact of the audio‐visual tool was likely to be more robust than that of the visual pamphlet (Figure 4).

According to the results of the LSD test, the Audio‐visual animation and visual pamphlet approaches had a significant impact on nurses' practice. The visual pamphlet, however, had a greater effect compared to the Audio‐visual animation method.

## 4 DISCUSSION

This study investigated the efficacy of audio‐visual animation and visual pamphlets in enhancing nurses' awareness, perspective, and practices regarding LBP prevention compared to a control group. Both interventions significantly improved these outcomes, yet distinct patterns emerged over time.

The visual pamphlet group demonstrated superior improvements in awareness and practice scores at both baseline and follow‐up. This aligns with research suggesting that static formats facilitate self‐paced learning and knowledge retention.^5^, ^11^, ^14^ Pamphlets' visual nature caters to diverse learning styles^15^ and reduces cognitive load,^16^, ^17^ likely contributing to their effectiveness in promoting practice changes. The visual pamphlet group demonstrated a more significant improvement in awareness and practice scores. This finding aligns with research by Xiao et al.,^14^ who suggest that static images in educational materials can facilitate flexible learning by allowing learners to revisit information at their own pace. This is particularly advantageous for reinforcing practical steps related to LBP prevention techniques that need to be integrated into daily routines.

This could be attributed to several factors. Pamphlets offer several advantages. First, their static format allows for self‐paced review, unlike fleeting video information.^11^ This repetition strengthens understanding and knowledge retention.^5^ Second, pamphlets cater to kinesthetic learners by visually depicting techniques.^15^ Finally, unlike video lectures, pamphlets reduce cognitive load and are readily accessible for just‐in‐time learning.^16^, ^17^ These format and accessibility benefits likely explain the improved practices observed in the pamphlet group.

In contrast, studies by Wardaningsih^18^ and Kim et al.^9^ highlight the effectiveness of audio‐visual animation interventions in enhancing knowledge and practical in various populations. While these studies focused on knowledge acquisition rather than practical implementation, they suggest a potential benefit for animation approaches in specific learning contexts.

While some argue that audio‐visual animations can overload working memory due to their richness,^19^ potentially hindering information retention, visual pamphlets offer a simpler presentation style. This aligns with Mayer's concept of reduced cognitive load, which may explain the observed improvement in practices with pamphlets over a 4‐week period.^20^, ^21^

However, an unexpected decline in perspective scores was observed in this group at follow‐up. While effective in knowledge transfer and skill acquisition, pamphlets may be less impactful in fostering long‐term perspective shifts. This unexpected finding suggests a potential limitation of this format.^22^ It is possible that the static nature of pamphlets may not be as effective in creating an emotional connection with the content, which is often crucial for inducing lasting changes in perspective. Further research is needed to explore the factors influencing perspective change over time and to identify strategies to enhance the attitudinal impact of pamphlets.

Audio‐visual animations exhibited consistent improvements across all outcomes, with a particularly pronounced increase in perspective scores. This supports research indicating the potential of multimedia interventions to enhance knowledge, attitudes, and perspectives.^23^ The dynamic and engaging nature of animations, as suggested by Zarifsaniey et al.,^21^ likely contributed to sustained changes in perspective by creating a more immersive and memorable learning experience.

The engaging nature of animations, with their combination of sound, visuals, and potentially interactive elements, might have fostered a more positive learning experience compared to the static format of the pamphlets. This suggests that animations may be particularly effective in influencing attitudes and fostering a more proactive approach to LBP prevention. However, the potential for cognitive overload remains a concern, as excessive stimuli can hinder learning.^24^, ^25^

The differential impact of the interventions on perspective is noteworthy. While pamphlets excelled in knowledge transfer and practical skill development, animations demonstrated a more enduring influence on attitudes. These findings suggest that a combined approach, incorporating elements of both methods, could optimize outcomes. For example, integrating short video clips into pamphlets or providing supplemental information within animations could enhance the overall effectiveness of the educational materials. Future research should explore the optimal sequencing and integration of these modalities, considering factors such as learner characteristics, content complexity, and learning objectives.

## 5 IMPLICATIONS AND FUTURE RESEARCH

These findings suggest that both visual pamphlets and audio‐visual animations offer valuable tools for LBP prevention education, but their effectiveness might vary depending on the targeted learning outcome (awareness, perspective, or practice). Future research could explore this concept further by incorporating larger and more diverse nurse populations. Additionally, extending the follow‐up period would provide insights into the long‐term efficacy of these educational methods.

In practice, healthcare institutions can leverage this knowledge to develop targeted educational programs. A combination of visual pamphlets and audio‐visual animations could cater to diverse learning styles among nurses, empowering them to choose the format that best suits their needs. Ultimately, this can contribute to a more knowledgeable and proactive nursing workforce, better equipped to prevent LBP and ensure their well‐being.

## AUTHOR CONTRIBUTIONS

**Nahid Zarifsanaiey**: Conceptualization; Writing—original draft; Methodology; Validation; Writing—review and editing; Software; Project administration; Data curation; Supervision. **Zahra Yazdani**: Investigation; Methodology; Validation; Visualization; Writing—review and editing; Formal analysis; Software; Data curation. **Zahra Karimian**: Investigation; Validation; Methodology; Conceptualization; Software; Project administration; Data curation; Writing—review and editing. **Hadi Raeisi Shahraki**: Methodology; Visualization; Formal analysis; Writing—review and editing.

## CONFLICT OF INTEREST STATEMENT

The authors declare no conflicts of interest.

## ETHICS STATEMENT

The study was carried out after receiving ethical approval from the local ethics committee of SUMS (1398.040) and coordinating with the teaching hospitals. The study objectives were explained to the participants, and written informed consent was obtained. To ensure anonymity, no names were used on the questionnaires, and a research assistant to prevent any errors encoded completed questionnaires. The participants were assured of the confidentiality of their data and that only aggregated statistics would be presented. The participants were also informed of their right to withdraw from the study at any time.

## TRANSPARENCY STATEMENT

The lead author Zahra Karimian affirms that this manuscript is an honest, accurate, and transparent account of the study being reported; that no important aspects of the study have been omitted; and that any discrepancies from the study as planned (and, if relevant, registered) have been explained.

## TAKE‐HOME MESSAGE

Low back pain (LBP) is recognized as an occupational disease. This study suggests that both visual pamphlets and audio‐visual animations can be effective ways to improve nurses' awareness, perspective, and practices regarding lower back pain prevention. These approaches prove valuable as suitable, cost‐effective, and flexible options, particularly for individuals with busy schedules.

## Data Availability

The datasets used and/or analyzed during the current study are available from the authors on request. All authors have read and approved the final version of the manuscript. The Corresponding author had full access to all of the data in this study and takes complete responsibility for the integrity of the data and the accuracy of the data analysis. The data that support the findings of this study are available from the corresponding author upon reasonable request.
